# Cadmium Speciation Distribution Responses to Soil Properties and Soil Microbes of Plow Layer and Plow Pan Soils in Cadmium-Contaminated Paddy Fields

**DOI:** 10.3389/fmicb.2021.774301

**Published:** 2021-12-03

**Authors:** Xiaodong Hao, Lianyang Bai, Xueduan Liu, Ping Zhu, Hongwei Liu, Yunhua Xiao, Jibiao Geng, Qianjin Liu, Lihua Huang, Huidan Jiang

**Affiliations:** ^1^Shandong Provincial Key Laboratory of Water and Soil Conservation and Environmental Protection, College of Resources and Environment, Linyi University, Linyi, China; ^2^Biotechnology Research Institute, Hunan Academy of Agricultural Sciences, Changsha, China; ^3^School of Minerals Processing and Bioengineering, Central South University, Changsha, China; ^4^College of Bioscience and Biotechnology, Hunan Agricultural University, Changsha, China

**Keywords:** Cd speciation, response characteristic, soil depth, Cd-contaminated paddy, microbial community

## Abstract

Cadmium (Cd) speciation ratio in arable land determines the Cd exposure risk and Cd uptake in crops. However, the driving mechanisms of Cd speciation change on the vertical scale of paddy fields remain poorly understood. In this study, the effects of plow layer and plow pan on Cd speciation distribution were investigated in a long-term Cd-contaminated rice ecosystem. The Cd accumulative effect within rice grain was enhanced with high levels of activated Cd speciation ratios in soils. Activated Cd speciation ratios were higher in plow layer soils, while stabilized Cd speciation ratios were elevated in plow pan soils. Soil physicochemical properties and soil microbes synergistically affected the Cd speciation changes in different ways between the two soil layers. Soil pH and organic elements in plow layer environment directly hindered the transformation of stabilized Cd speciation, while in plow pan environment, soil pH and organic elements indirectly decreased activated Cd speciation ratios and resulted in the accumulation of stabilized Cd speciation *via* regulating the predominant bacterial taxa. This study will improve our understanding of how soil environments regulate Cd speciation distributions in rice ecosystems and help to seek effective remediation methods of Cd-contaminated paddy fields to reduce the Cd accumulation in rice.

## Introduction

Cadmium (Cd), a highly toxic and carcinogenic heavy metal species, has attracted substantial attention from researchers (Aitio and Tritscher, 2004; Hu et al., 2020). The Cd contamination of paddy fields causes soil degeneration and cereal quality degradation, raising serious food supply, and food security concerns (Rafiq et al., 2014; Jiang et al., 2020). Rice (*Oryza sativa* L.), as the main food for more than half the global population, readily assimilates Cd *via* root uptake (Khan et al., 2017; Shi et al., 2020). Consumption of Cd-contaminated rice enhances the dietary exposure to Cd and threatens human health (Santeramo and Lamonaca, 2021). Consequently, Cd-contaminated paddy soils are in urgent need of remediation to reduce the Cd bioaccumulation of crops and adverse health effects (Huang et al., 2019). This requires a clear understanding of the biogeochemical behavior of Cd and Cd speciation change controlled by various soil environments (Li et al., 2021).

Generally, Cd deposited on soils exists as the complexes precipitated with soil components, such as carbonate, Fe-Mn oxides, organic matter, and primary minerals. Cd speciation can be divided into acid-soluble, reducible, oxidizable, and residual fractions based on sequential extraction procedures developed by the European communities bureau of reference (BCR; Gleyzes et al., 2002). The acid-soluble and reducible fractions are considered to be bioavailable for plants, but the oxidizable and residual fractions of Cd are the recalcitrant chemical forms. The Cd assimilation of crops and remediation efficiency of Cd pollution not only depend on the total Cd content in soils, but also more strongly on the distributional characteristics of Cd speciation, especially on the vertical scale (Chen et al., 2019). The plow layer is the surface soil region of crop growth that is characterized by the rich soil nutrients, loose aggregate structure, and root dense zone. The soil properties in this layer are constantly affected by the agricultural management and natural factors. The plow pan is recognized as the oligotrophic, anoxic, and sparse root soil layer, and is relatively far from the interference of farming practices. However, some soil remediation methods and agricultural production activities like deep tillage, topsoil removal, and soil dilution can alter the soil properties throughout the soil profile, and thereby affect the spatial distribution of Cd and Cd accumulation in plants (Peng et al., 2018). Previous investigations have focused on the spatial distribution and risk assessment of total Cd in a variety of habitats (Li and Ji, 2017; Deng et al., 2019), while Cd speciation differences along the vertical profile and their driving mechanisms are largely ignored.

The changes in soil environmental conditions are associated with the complex and diverse Cd speciation distribution. Soil pH can affect the activity of soil microorganisms and the synthesis of organic matter, changing the equilibrium point of Cd precipitation-dissolution. Soil redox potential can be highly mediated by soil moisture, shaping the sorption/desorption dynamics of Cd bound to Fe-Mn oxides and sulfides (Li et al., 2020a). Soil organic ligand concentration plays an important role in the transformation of different Cd forms in soils (Wu et al., 2018). The Cd speciation can also be transformed through a biogeochemical process, changing the Cd mobility, toxicity, and bioavailability. Chemoautotrophic bacteria, fungi, and the mixture culture of acid-tolerant microorganisms have been used for the biomobilization of Cd compounds through the direct and indirect mechanisms (Li et al., 2020b). The biological methods of Cd bioimmobilization, such as adding microorganism directly, microbial preparation, and biostimulation, can transform toxic Cd compounds into low- or non-toxic states. Although the alterations of soil microbial communities and soil properties in different soil depths are studied extensively (Müller et al., 2016; Anderson et al., 2017; Yu et al., 2021), little has been done to address the significant question concerning the association of Cd speciation change with the diverse soil environments of the plow layer and plow pan soils in Cd-contaminated paddy fields (Zhang et al., 2021). Likewise, the underlying mechanisms of Cd speciation distribution regulated by the composite effects of soil properties and soil microbes remain unclear and need further study. These results will provide a comprehensive understanding of Cd speciation distributions and their driving factors responding to the plow layer and plow pan soils of paddy field systems, and further help to remediate Cd-contaminated paddy fields efficiently (Fonti et al., 2016).

In this study, we analyzed the Cd speciation distribution characteristics between plow layer and plow pan soils in long-term Cd-contaminated paddy fields. The aims of this study were to evaluate the responses of Cd speciation distribution to soil properties and soil microbes, and finally to reveal the transformation mechanisms of Cd speciation regulated by the plow layer and plow pan soil environments.

## Materials and Methods

### Site Description and Sample Collection

The soil sampling site was located in a rice-growing region with a subtropical monsoon humid climate in Xiangtan County, Hunan Province, Southeast China (27°77′ N′112°88′ E; Supplementary Figure 1A). The mean annual temperature in this paddy area was 16.7°C–18.3°C, with mean annual precipitation of 1,300mm. The rice fields were adjacent to a steel smelter factory and had been contaminated by irrigation water mixed with Cd-containing industrial wastewater for about 10years. For our study, in rice mature period, a total of 15 sampling points covering an approximate area of 1.5 hectares were selected randomly (Supplementary Figure 1B) in October 2020. Rice samples of indica rice cultivar (Yuzhenxiang, 15 samples) and paired bulk soil samples (30 samples) including plow layer soils (0–20cm) and plow pan soils (20–40cm) were collected using a T-sampler. Plant residues and rocks in each soil sample were removed by a 2-mm nylon mesh. These soil samples were taken back to the laboratory and stored at 4°C or −20°C for the soil physicochemical measurements or microbial community analysis, respectively.

### Soil Property Analysis

Rice grain and soil samples were air-dried and milled to power with size of <150μm using the multi-purpose disintegrator (DE-250, Rhodiola Instrument, Zhejiang, China). Rice grain samples (0.2g) were digested by an electric heating plate (XJS20-42, Laboratory Instrument, Tianjin, China) using the HNO3 and HClO4 (5:1, vol/vol). Soil samples (0.2g) were digested with an acid mixture of HNO3, HF, and HClO4 (10:5:2, vol/vol). Total Cd contents in rice grain, soil heavy metals, total P, and total K contents were analyzed by ICP-OES (Optima 5300DV, PerkinElmer, Shelton, United States). Soil pH and oxidation reduction potential values (ORP, vs. Ag/AgCl) were analyzed at a soil-to-water ratio of 1:2.5 (wt/vol) and measured by a pH meter (BPH-220, Bell Instrument, Dalian, China). Soil temperature (Tsoil) was measured immediately during the soil sampling process by a thermometer (TR-6, Shunkeda, Guangzhou, China). Soil moisture was analyzed after drying for 24h at 80°C using the fresh soil samples. Organic element (organic N, organic C, organic H, and organic S) contents were determined using an Elemental Analyzer (Elementar Vario EL III, Elementar Analysensysteme, Hanau, Germany).

Cd speciation analysis of soil samples was carried out according to the sequential extraction procedure as described previously (Hao et al., 2019). Briefly, (i) the acid-soluble fraction (F-Aci) of Cd in soil samples (0.5g) was extracted at room temperature for 16h with 20ml of 0.1M acetic acid solution (pH 5.0) with continuous agitation; (ii) the reducible fraction (F-Red) of Cd in residue from (i) was leached with 20ml of 0.5M NH2OḤHCl (pH 1.5) at room temperature for 16h; (iii) the oxidizable fraction (F-Oxi) of Cd was extracted from the residue (ii) through adding 10ml of 8.8M H2O2 and was heated to 87±2°C for 1h with occasional agitation. A second 10ml aliquot of 8.8M H2O2 was then added and the sample was heated again to 87±2°C for 1h with intermittent agitation. After cooling, 20ml of 1.0M NH4OAc adjusted to pH 2.0 with HNO3 was added and shaken at room temperature for 16h; and (iv) the residual fraction (F-Res) of Cd in residue (iii) was digested with a HNO3-HF-HClO4 mixture as described for the total metal analysis. Between successive extractions, the supernatant was collected by centrifugation at 3600g for 20min. We used the F-Aci ratio, F-Red ratio, F-Oxi ratio, and F-Res ratio representing the Cd speciation transformation percentages toward the acid-soluble fraction, reducible fraction, oxidizable fraction, and residual fraction of Cd, respectively. The calculation formula is as follows: Cd speciation ratio (%)=C1/C2×100, where C1 represents the contents of each Cd speciation, and C2 represents the total Cd contents of soil samples.

### Microbial Community Analysis

#### DNA Extraction, PCR Amplification, High-Throughput Sequencing

Total genomic DNA was extracted from 1.0g of each soil sample using the E.Z.N.A. Soil DNA kit (Omega Bio-Tek Inc., United States) according to the manufacturer’s protocol. DNA concentration and quality were detected by NanoDrop ND-1000 Spectrophotometer (NanoDrop Technologies) using the ratios of absorbance at 260/280nm and 260/230nm. Soil microbial biomass was represented by the amount of microbial DNA in soils, which was assessed by the percentage of DNA mass extracted from soils compared to the dried soil weight (Sanaullah et al., 2016).

Paired primers of 515F (5′-GTGCCAGCMGCCGCGGTAA-3′) and 806R (5′-GGACTACHVGGGTWTCTAAT-3′) with unique barcode sequences were used to amplify the V4 hypervariable region of 16S rRNA genes. PCR amplification was performed in 25μl reactions containing 12.5μl 2×Taq PCR Master Mix (Vazyme, Piscataway, United States), 1μl of template DNA, 1μl (10nm) of each primer, and 9.5μl of ddH_2_O water. Thermal cycling procedures were initial denaturation at 94°C for 5min, followed by 30cycles of 94°C for 45s, 62°C for 45s, and 72°C for 30s, with final extension at 72°C for 10min. PCR products were purified by 1.2% (wt/vol) agarose gel and recovered using an E.Z.N.A. TM Gel Extraction Kit (Omega Bio-Tek Inc., United States). PCR amplicons in each sample were pooled in equimolar concentrations, and high-throughput sequencing was performed on the Illumina Miseq platform (Miseq PE250).

#### Data Processing

Raw 16S rRNA amplicon sequencing data were assigned to each sample according to the unique barcode sequences using the Galaxy Illumina sequencing pipeline.^1^ Forward and reverse primers reads were trimmed with up to 1.5 mismatches allowed. The Btrim program was used to remove the unqualified sequences with a threshold of QC>20 over a 5bp window size (Kong, 2011), and then, pair-ended sequences were combined and quality filtered by Flash (Magoč and Salzberg, 2011). Operational taxonomic units (OTUs) were clustered by UPARSE with a sequence threshold of 97% similarity (Edgar, 2013). Taxonomic assignment of 16S representative sequences was performed based on the ribosomal database project (RDP) classifier with a minimum of 50% confidence estimates (Wang et al., 2007). The raw 16S rRNA sequencing data were deposited in the NCBI Sequence Read Archive database (SRP291811).

#### Functional Prediction

The phylogenetic investigation of communities by the reconstruction of unobserved states (PICRTUSt) was used to predict the abundance of gene families in environmental communities using the 16S rRNA sequencing data of plow layer and plow pan soil samples (Langille et al., 2013). The Kyoto encyclopedia of genes and genomes (KEGG) database and closed reference OTUs based on the Greengenes reference taxonomy (Greengenes 13.5) were used to predict the molecular functions of each soil sample. The 16S copy number was normalized, and then, molecular functions were predicted and final data were summarized into KEGG pathways.

#### Network Construction

Phylogenetic molecular ecological networks (pMENs) were constructed by sequencing of 16S rRNA gene amplicons based on random matrix theory (RMT; Deng et al., 2012). The OTUs detected in at least 12 out of 15 replicates were employed to ensure correlation reliability for network analysis. The pMENs of plow layer and plow pan soil samples were constructed and analyzed on the IEG MENA Pipeline,^2^ and the network plots were visualized in Cytoscape 3.6.0 software. The network was split into different modules to represent the modularity property. Sub-networks of plow layer and plow pan soil samples were also built separately using the F-Aci ratio, F-Red ratio, F-Oxi ratio, and F-Res ratio as microbial factors to analyze the correlation between the Cd speciation ratio and relevant OTUs.

### Statistical Analysis

In this study, the Shannon index was calculated to measure the biodiversity of the microbial community in plow layer and plow pan soils using the resampled 16S OTU subsets (10, 000 sequences per soil sample). A Venn diagram was constructed using “Venny 2.1.”^3^ Detrended correspondence analysis (DCA) was used to present the bacterial community structure heterogeneity between the two soil layers. Dissimilarity analyses of MRPP, ANOSIM, and Adonis were conducted to test the differences in microbial structure in the plow layer and plow pan soil samples. Redundancy analysis (RDA) and variation partitioning analysis (VPA) were performed to estimate the contributions of environmental variables to the bacterial community using R vegan package. A paired-sample *t*-test was employed to identify the differences in the soil physicochemical properties, relative abundance of dominant microbes and selected pathways, soil DNA concentrations, and Shannon index between the two soil layers using IBM SPSS Statistics 21.0 software. The correlation analysis between Cd accumulation contents of rice grain, soil parameters, soil microbes, and Cd speciation ratios was performed using a Pearson correlation test. A partial least square path model (PLSPM) using the “amap,” “shape,” “diagram,” and “plspm” packages in R was used to show the effects of soil factors and microbial community structures on Cd speciation distribution in plow layer and plow pan soils.

## Results

### Effects of Soil Properties on Cd Speciation Distribution

Total heavy metal contents showed significant differences between plow layer and plow pan soil samples (Table 1). The total Cd, Cr, Cu, Pb, and Zn contents in plow layer soils were significantly (*p*<0.01) higher than those in plow pan soils, which was mainly attributed to the artificial exogenous introduction and adsorption of heavy metals in plow layer soils. However, only the Cd contents in both plow layer (19.0mg/kg) and plow pan (0.4mg/kg) soils exceeded the Environmental Quality Standard for Soils of China (EPM, 2018) for Cd (0.3mg/kg) in paddy fields. The mean Cd content in rice grain was 2.9±0.8mg/kg, which was 14.5 times higher than the Cd standard threshold (0.2mg/kg) of National food safety standards of China. Moreover, the contents of total Mn, total K, and soil pH values decreased, while soil moisture, soil temperature, total P, and organic elements contents (organic N, organic C, and organic H) increased in plow layer soils. Soil ORP and organic S contents showed no significant (*p*>0.05) differences between these two soil layers.

**Table 1:** Physicochemical properties (means ± standard deviation, *n*=15) analysis in the plow layer and plow pan soils.

Item	Plow layer	Plow pan	Item	Plow layer	Plow pan
Total Cd, mg/kg	**19.0±11.1**	0.4±0.2	Tsoil,°C	**12.7±1.0**	11.9±0.4
Total Cr, mg/kg	**124.5±21.7**	111.8±16.5	ORP, mV	315.9±17.7	311.6±17.9
Total Cu, mg/kg	**21.2±2.5**	15.1±3.3	Total P, mg/kg	**557.3±119.5**	442.3±89.8
Total Pb, mg/kg	**27.8±12.2**	17.9±8.9	Total K, g/kg	10.0±0.5	**10.7±0.5**
Total Zn, mg/kg	**182.6±63.0**	54.1±6.0	Organic N,%	**0.20±0.04**	0.07±0.02
Total Mn, mg/kg	304.1±110.9	**697.5±595.2**	Organic C,%	**2.2±0.5**	0.6±0.3
pH	6.3±0.4	**7.4±0.3**	Organic H,%	**0.81±0.07**	0.67±0.10
Moisture,%	**50.0±6.9**	24.5±4.8	Organic S,%	0.03±0.01	0.02±0.01

*Quantitative values in bold font indicate the significantly higher means in the plow layer or plow pan soils (paired-sample t-test, p<0.05)*.

**Figure 1:**
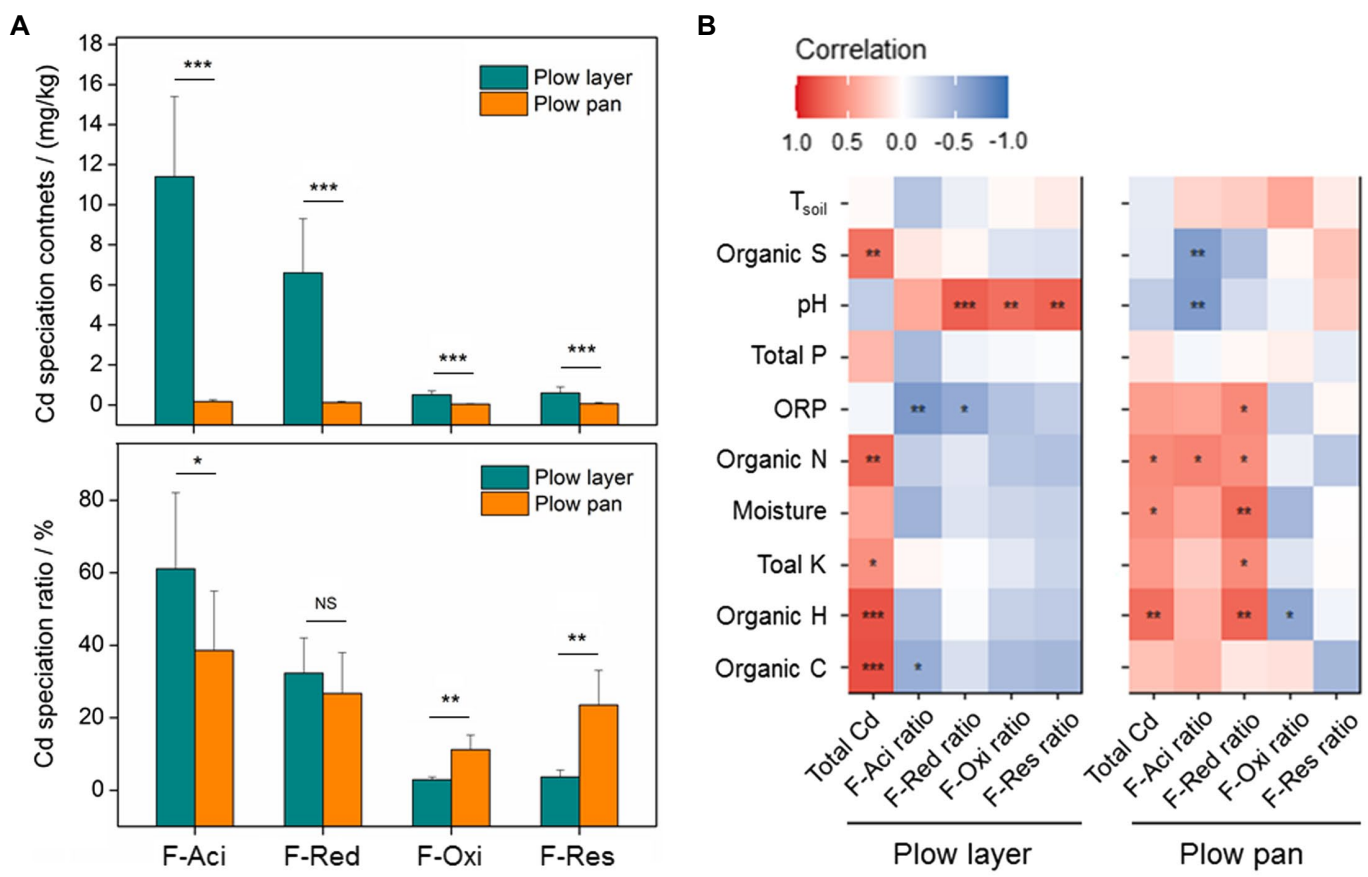
Cd speciation distribution and relationships between the soil variables and Cd speciation ratios in the plow layer and plow pan soils. **(A)** Distributions of Cd speciation contents and ratios between the two soil layers. **(B)** Pearson correlation analysis. F-Aci, F-Red, F-Oxi, and F-Res indicate the acid-soluble fraction, reducible fraction, oxidizable fraction, and residual fraction of Cd, respectively. Asterisks indicate the significant difference or correlation (^*^p<0.05, ^**^p<0.01, and ^***^p<0.001).

Similarly, the contents of each Cd species were also significantly higher (*p*<0.001) in plow layer soils than in plow pan soils, but differences in each Cd speciation ratio in the two soil layers were observed (Figure 1A). The acid-soluble fraction (F-Aci) and reducible fraction (F-Red) of Cd with high bioavailability were the dominant fractions of Cd speciation in these two soil layers, but the F-Aci ratio in plow layer soils (61.1%) was significantly higher (*p*<0.05) than in plow pan soils (38.6%). However, the ratios of the oxidizable fraction (F-Oxi, 11.2%) and residual fraction (F-Res, 23.5%) in plow pan soils were significantly (*p*<0.01) increased compared to those in plow layer soils (2.9 and 3.7%, respectively). Pearson correlation analysis showed that the Cd accumulation contents of rice grain positively correlated with F-Aci ratio and F-Red ratio in plow layer soils (*p*<0.05), whereas negatively correlated with F-Res ratio in plow pan soils (*p*<0.01; Table 2), which indicated that activated Cd speciation ratios in soils enhanced the Cd accumulative effect in rice grain. Total Cd content had a significantly positive correlation with organic elements (*p*<0.01) and total K (*p*<0.05) in plow layer soils, and a positive correlation with soil moisture, organic N, and organic H (*p*<0.05) in plow pan soils (Figure 1B). For the Cd speciation ratio in the plow layer, soil pH indicated a significantly (*p*<0.01) positive correlation with the ratios of F-Red, F-Oxi, and F-Res, but a negative correlation with the F-Aci ratio (*p*<0.01) in the plow pan. Moreover, soil moisture, total K, organic N, and organic H had a significantly positive correlation (*p*<0.01) with the F-Red ratio of plow pan soils. These results showed that the differences in soil properties, especially soil pH and organic elements, distinctly impacted the Cd speciation distribution between the plow layer and plow pan soils. As a result, more activated Cd species (F-Aci and F-Red fractions) were present in plow layer soils, while more stabilized Cd species (F-Oxi and F-Res fractions) were present in plow pan soils.

**Table 2:** Pearson correlation between Cd accumulation contents in rice grain and Cd speciation ratios in the plow layer and plow pan soils.

Item	Plow layer		Plow pan		Coefficient	*P*	Coefficient	*P*
Total Cd, mg/kg	0.225	0.420	0.381	0.161
F-Aci ratio,%	0.573	0.026^*^	0.136	0.630
F-Red ratio,%	0.569	0.027^*^	0.171	0.543
F-Oxi ratio,%	0.481	0.070	0.075	0.791
F-Res ratio,%	−0.465	0.081	−0.716	0.003^**^

*Asterisks indicate the significant correlation*

* *p<0.05;*

** *p<0.01.*

### Phylogenetic Composition and Soil Bacterial Communities

**Figure 2:**
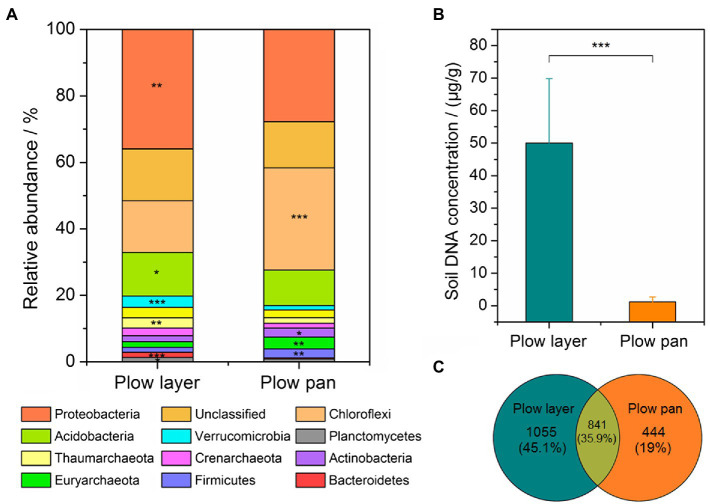
Comparison of the bacterial community composition and microbial mass in the plow layer and plow pan soils. **(A)** Dominant microbes at the phylum level. **(B)** Variations in the soil DNA concentrations. **(C)** Variations in the OTU numbers. Asterisks indicate the significant difference (paired-sample *t*-test, ^*^p<0.05, ^**^p<0.01, and ^***^p<0.001).

Microbial community differences in plow layer and plow pan soils were detected by 16S rRNA amplicon sequencing. The soil 16S rRNA sequences were assigned to 35 bacterial phyla. The top three dominant phyla, accounting for more than 53% of the relative abundance, were *Proteobacteria* (plow layer 36.0% and plow pan 11.6%), *Chloroflexi* (15.5 and 30.8%, respectively), and *Acidobacteria* (13.1 and 10.6%, respectively), which were significantly different (paired-sample *t*-test, *p*<0.05) between these two soil layers (Figure 2A). The relative abundances of *Verrucomicrobia*, *Thaumarchaeota*, *Crenarchaeota*, and *Planctomycetes* in plow layer soils were significantly (*p*<0.05) higher than in plow pan soils, but *Actinobacteria*, *Euryarchaeota*, and *Firmicutes* were significantly (*p*<0.05) lower. Furthermore, the DNA concentration in plow layer soils (50.0μg/g soil) was much (*p*<0.001) higher than that in plow pan soils (1.2μg/g soil; Figure 2B). Meanwhile, there were more unique OTUs in plow layer soils (1,055, 45.1%) than in plow pan soils (444, 19.0%; Figure 2C), indicating the higher microbial mass in plow layer soils.

**Figure 3:**
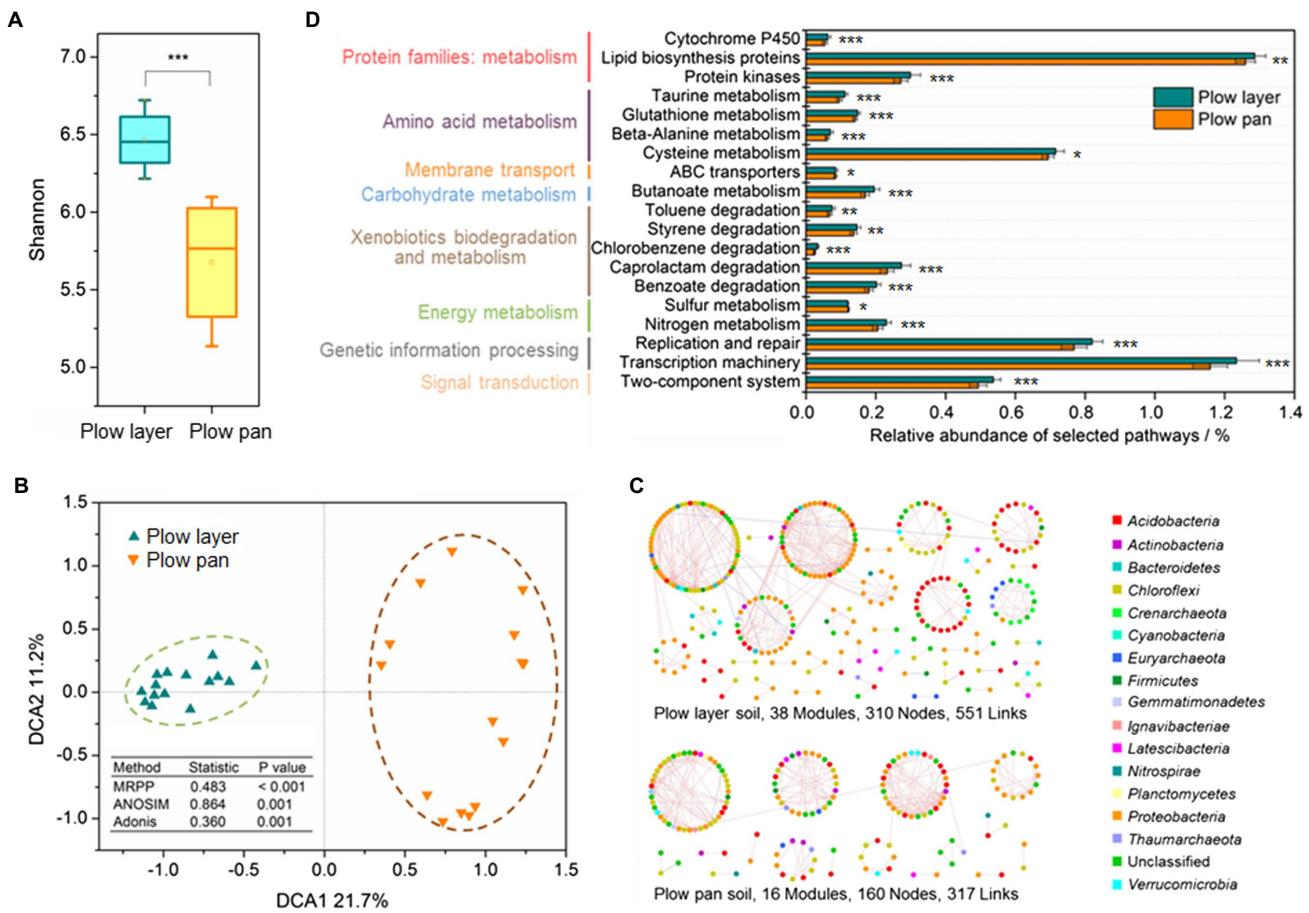
Diversity and structure of bacterial communities in the plow layer and plow pan soils. **(A)** Shannon index. **(B)** DCA analysis. **(C)** Molecular ecological networks. **(D)** Functional predictions. Asterisks indicate the significant difference (paired-sample *t*-test, ^*^p<0.05, ^**^p<0.01, and ^***^p<0.001).

Shannon index was used to distinguish the variations of α-diversity in plow layer and plow pan soil samples (Figure 3A). Shannon indexes from plow layer soils were significantly (paired-sample *t*-test, *p*<0.001) increased compared to those of plow pan soils. Pearson correlation analysis indicated that there were no significant correlations between the Shannon index and Cd speciation ratio in plow layer and plow pan soils (Supplementary Table 1). The β-diversity measurements also showed significant divergence between these two soil layers. DCA revealed that the microbial community structure of plow layer soils tended to be more similar, but observably distinct from that of plow pan soils (Figure 3B). Dissimilarity tests using MRPP (*p*<0.001), ANOSIM (*p*=0.001), and Adonis (*p*=0.001) further confirmed this differentiation of soil bacterial communities between plow layer and plow pan soils.

Phylogenetic molecular ecology networks (pMENs) were constructed to explore the microbial interactions in plow layer and plow pan soils. The microbial pMEN in plow layer soils (38 modules, 310 nodes, and 551 links) was larger and more complex than that in plow pan soils (16 modules, 160 nodes, and 317 links), indicating that the microbiome in the plow layer had more species interactions (Figure 3C). The plow layer group had an average path distance of 8.305, which was higher than that of the plow pan group (4.089), demonstrating closer microbial connectivity in plow layer soils (Supplementary Table 2). The molecular functions of each soil sample were predicted using PICRUSt as a predictive exploratory tool. The functional classification and most basic metabolic pathways of level II orthology groups (KOs in KEGG) between plow layer and plow pan soil samples were consistent (Supplementary Figure 2). However, several different pathways between these two soil layers were still observed (Figure 3D). The cell metabolic functions from plow layer soil samples consistently showed higher relative abundances than those of plow layer soils (paired-sample *t*-test, *p*<0.01), including the protein families: metabolism, amino acid metabolism, carbohydrate metabolism, and energy metabolism. In addition, some xenobiotics biodegradation and metabolism pathways also showed higher abundances in plow layer soils (*p*<0.01).

### Relationship Between Soil Microbes and Cd Speciation Distribution

**Figure 4:**
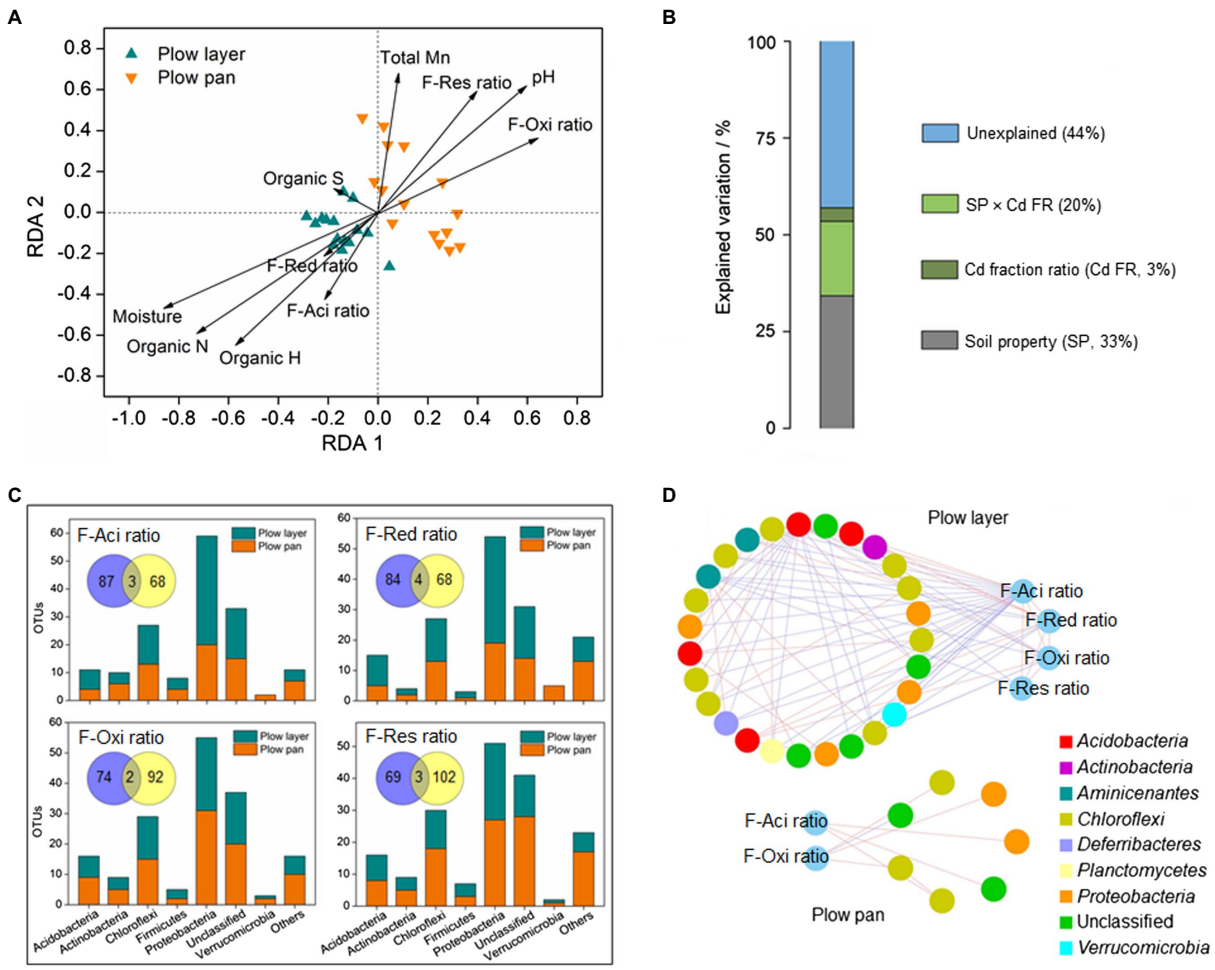
Distinct relationships between the soil microbial community and Cd speciation ratios in the plow layer and plow pan soils. **(A)** Redundancy analysis. **(B)** Variation partitioning analysis. **(C)** Pearson correlation analysis. **(D)** Sub-network analysis. F-Aci, F-Red, F-Oxi, and F-Res indicate the acid-soluble fraction, reducible fraction, oxidizable fraction, and residual fraction of Cd, respectively. Venn diagrams represent the OTU numbers related with the Cd speciation ratio between the plow layer soils (blue) and plow pan soils (yellow).

There were great differences in microbial communities between plow layer and plow pan soils (Figures 2, 3), and these differences obviously affected the Cd speciation distribution in the two soil layers (Figure 4). Redundancy analysis (RDA) showed that the microbial community of plow layer soils had a positive relationship with the F-Aci ratio, F-Red ratio, soil moisture, and organic elements (Figure 4A). However, the plow pan soil bacterial community was positively associated with the F-Oxi ratio, F-Res ratio, soil pH, and total Mn. Variation partitioning analysis (VPA) was performed to determine the relative contribution of environmental variables on the bacterial community (Figure 4B). The soil Partial least square path modelroperties and Cd speciation ratio could explain 33 and 3% of variation in the soil bacterial communities, respectively. Their interaction explained 20% of variation, leaving 44% of the variation unexplained. These results showed that the soil microbiome might facilitate the Cd speciation transformation process to F-Aci and F-Red fractions in plow layer soils, and to F-Oxi and F-Res fractions in plow pan soils. Moreover, the relative abundance of some heavy metal metabolism-related molecular functions, such as ABC transporters, sulfur metabolism, and cysteine metabolism, was higher (*p*<0.05) in plow layer soils (Figure 3D), also indicating the roles of soil microbes in maintaining more activated Cd speciation in plow layer soils compared to plow pan soils.

Pearson correlation analysis revealed the OTU numbers associated with the variations of each Cd speciation ratio between the two soil layers (Figure 4C). There were more unique OTUs correlated with the F-Aci ratio and F-Red ratio in plow layer soils (87 and 84, respectively) than plow pan soils (68 and 68, respectively). In contrast, the number of unique OTUs correlated with the F-Oxi ratio and F-Res ratio in plow pan soils (92 and 102, respectively) was higher than that in plow layer soils (74 and 69, respectively). Most of the OTUs associated with the F-Aci ratio and F-Red ratio belonged to the dominant phylum-level microbes of *Proteobacteria* and *Acidobacteria* in plow layer soils. However, the major OTUs associated with the F-Oxi ratio and F-Res ratio were the dominant phyla of *Chloroflexi* and *Actinobacteria* in plow pan soils. The sub-networks of plow layer and plow pan soil bacterial communities using each Cd speciation ratio as microbial factors were constructed to detect the interactions between the Cd speciation ratio and related OTUs (Figure 4D). A more closely connected sub-network was observed in the plow layer compared with the plow pan. There were 27 OTU nodes linking to four Cd speciation ratios (40 links) in plow layer soils, but only seven OTU nodes linked to F-Aci and F-Oxi ratios (seven links) in plow pan soils. Meanwhile, there were 31 links (77.5%) between the OTU nodes and F-Aci and F-Red ratios in plow layer, while four links (57.1%) associated with the F-Oxi ratio were found in the plow layer. These results indicated that the related OTUs exhibited more cooperation to mediate the activated Cd speciation transformation (F-Aci and F-Red ratios) in plow layer soils, and the soil bacteria of plow pan soils were more inclined to participate in the formation of stabilized Cd speciation (F-Oxi and F-Res ratios).

### Linkages Between Soil Factors, Microbial Community Structure, and Cd Speciation Distribution

**Figure 5:**
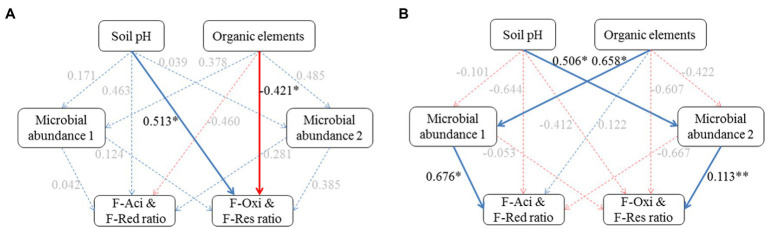
Effects of soil factor and microbial community composition on Cd speciation ratios in the plow layer and plow pan soils. **(A)** Plow layer. **(B)** Plow pan. Blue lines and red lines indicate the positive and negative correlations, respectively, and dashed lines indicate the correlations are not at a significant level. Asterisks indicate the significance level: ^*^p<0.05, ^**^p<0.01, and ^***^p<0.001.

Partial least square path models (PLSPM) were constructed to investigate the pathways of the impact of soil environments on Cd speciation distribution between plow layer and plow pan soils (Figure 5). The goodness-of-fit of the modeling in the plow layer and plow pan soils was 0.6103 and 0.6085, respectively. In plow layer soils, soil pH and organic elements were identified as the most significant factors (*p*<0.05) with directly positive and negative relationships to the F-Oxi and F-Res ratios, respectively (Figure 5A). In plow pan soils, soil pH and organic elements had positive associations with microbial abundance 1 of *Acidobacteria* and *Proteobacteria* and microbial abundance 2 of *Actinobacteria* and *Chloroflexi*, which were positively related to the F-Aci and F-Red ratios, and the F-Oxi and F-Res ratios, respectively (Figure 5B). The PLSPM results revealed that the low pH values and high organic element contents in plow layer soils limited the formation of the F-Oxi and F-Res fractions and thus maintained higher F-Aci and F-Red ratios. In comparison with plow layer soils, the effects of high soil pH and low organic elements in plow pan soils significantly (*p*<0.05) regulated the relative abundance of dominant phylum-level microbes to mediate the Cd speciation shifts and thereby decrease the F-Aci and F-Red ratios and increase the F-Oxi and F-Res ratios in plow pan soils.

## Discussion

In this study, we comparatively investigated the Cd speciation distribution between plow layer and plow pan soils in Cd-contaminated paddy fields and their responses to environmental variables and microbial communities. The results demonstrated that the soil environment of the plow layer maintained higher ratios of activated Cd speciation, while the plow pan soils mediated more stabilized Cd speciation formation. The Cd speciation distribution mechanisms regulated by soil physicochemical properties and soil microbes in two soil layers were different.

Distinct differentiations of total Cd, Cd speciation, and other soil physicochemical properties were observed in plow layer and plow pan soils (Table 1, Figure 1A). The changes in soil factors caused by farming practices altered the Cd distributions. Polluted river irrigation, straw turnover, and Cd-bearing fertilizer application resulted in the increased contents of total Cd, total P, and organic elements in plow layer soil. Water-retaining properties and Cd bound to plant residues and oxide minerals restricted the downward migration of Cd to plow pan soils (Matos et al., 2001; Dong et al., 2016), which was in accordance with the findings of significantly higher total Cd contents in plow layer soils and their positive correlation with organic elements (*p*<0.01; Table 1B). Pearson correlation analysis indicated that soil factors, especially soil pH and organic elements, significantly affected the Cd speciation distribution between the two soil layers (Figure 1B; Wang et al., 2020). The excessive utilization of chemical fertilizers and rice straw decomposition decreased the soil pH value. A previous study showed that soil pH reduction significantly increased Cd bioavailability while reducing the pH from 7 to 6 (Zhang et al., 2019). In this study, the pH value in plow layer soils (6.3) was significantly decreased (*p*<0.05) than in plow pan soils (7.4). Under low pH conditions, the produced positive charges could competitively adsorb soil organic substances and metallic oxides with Cd^2+^. Meanwhile, low pH favored the Cd release associated with soil organic matters, which increased the Cd activity although there were higher organic element contents in plow layer soils (Jia et al., 2021). In addition, soil pH and organic matter were also important soil variables regulating the microbial growth and Cd complexation behaviors in different soil layers, and thereby influenced the microbial roles in the Cd speciation change (Jacob et al., 2018).

After that, 16S rRNA amplicon sequencing was performed to evaluate the alterations of soil microbial communities between plow layer and plow pan soils. The main composition of bacterial taxa was highly conserved, but differentiation in the relative abundance of dominated microbes was recorded between the two soil samples (Figure 2A). The bacterial phyla of *Proteobacteria* and *Acidobacteria*, and *Chloroflexi* and *Actinobacteria* were dominant in plow layer and plow pan soils, respectively. Compared with plow pan soil environments, the sufficient nutrients, energy sources, and oxygen-rich conditions in plow layer soils supported the increased microbial mass, Shannon index, and a variety of cellular metabolic functions, dramatically altering the microbial communities of the two soil layers (Figures 2, 3). The results of DCA showed that the phylogenetic β-diversities of the two microbial communities were distinctly different, although a large amount of inter-individual variation was found in plow pan soils compared with plow layer soil samples. Agricultural practices, such as fertilization, could serve to homogenize the soil micro-sites. This might explain why we observed more similar microbial community structures in plow layer soil samples (Liang et al., 2020). Additionally, distinctive network topologies were also observed at two soil sites (Figure 3C; Supplementary Table 2). The higher modularity and avgK value in the plow layer soil network both indicated that the topsoil bacterial community was more resistant and less influenced by disturbances, such as high heavy metals and toxic pollutants, than plow layer soils based on network theory (Saavedra et al., 2011), which was consistent with the findings that xenobiotic bioremediation-related pathways showed higher abundance in plow layer soils.

The shifts in the soil microbial community mediated the Cd speciation ratio distribution in the two soil layers. VPA showed that the changes between bacterial community and Cd speciation ratio were interrelated (Figure 4B). Redundancy analysis demonstrated that the microbial communities of plow layer and plow pan soils were positively associated with the transformations of available Cd speciation (F-Aci and F-Red ratios) and steady Cd speciation (F-Oxi and F-Res ratios), respectively, suggesting that the microbial communities in these two soil samples induced disparate Cd speciation changes (Figure 4A). The molecular function prediction analysis further verified the roles of the plow layer soil microbiome in promoting the formation of activated Cd speciation (Figure 3D). The plow layer soil bacteria with higher percentages of cysteine metabolism and the ABC transporter system could form Cd-metallothionein complexes and combined ATP-hydrolysis to reduce the microbial immobilization of Cd (Duong et al., 1996; Valls et al., 2000). In addition, the topsoil microbes possessing rich organic matter and the ability to metabolize sulfur could oxidize the Cd fractions bonding to humus and sulfide substrate into soluble Cd fractions, and thereby increase Cd activity in plow layer soils (Cao et al., 2018). Pearson correlation analysis demonstrated that many more unique OTUs mediated the Cd speciation transformation of the F-Aci and F-Red fractions, and these OTUs were mainly the members of *Proteobacteria* and *Acidobacteria* in plow layer soils. In plow pan soils, most of the OTUs participated in the formation of F-Oxi and F-Res fractions, which were the dominant phyla of *Chloroflexi* and *Actinobacteria*. The phylum *Acidobacteria*, with high relative abundance in plow layer soils, was sensitive to changes in pH and preferred relatively acidic soil conditions, which coincided with the low plow layer soil pH values (Mendes et al., 2015). Several members of *Acidobacteria* are able to degrade complex biopolymers (Conradie and Jacobs, 2021), probably facilitating the transformation of mobile Cd speciation. *Proteobacteria* are regarded as the most stress-tolerant microbes in heavily contaminated soils (Wang et al., 2019). The members belonging to *Proteobacteria* exhibited multiple nutrient classifications and microbial functional diversities. It was reported that multiple heavy metal oxidase genes encoded by *Proteobacteria* were involved in the resistance and transform of heavy metals. The decreasing tendency of *Chloroflexi* along the soil depth was consistent with a previous study (Sagova-Mareckova et al., 2016), which showed the facultative anaerobic members of *Chloroflexi* were widely detected in relatively oxygen poor conditions (Hartmann et al., 2010). They were associated with the degradation of organic compound pollutants, producing more functional group-rich metabolites, and transformed the exchangeable Cd fraction into more stable organic-bound forms. The phylum *Actinobacteria* favored low moisture and alkalescent soil environments, resulting in a higher abundance in plow pan soils. Soil Actinomycetes could generate abundant secondary metabolites and reduce Cd bioavailability through complexation, adsorption, and reduction (Sun et al., 2021).

Although both soil variables and soil microbial communities had apparent effects on Cd fraction changes, different mechanisms regulating Cd speciation change between the two soil layers were observed (Figure 5). The results of PLSPM revealed that in plow layer soils the effect of soil variables was higher than that of soil microbial communities, which suggested that soil pH and organic elements were more important than dominant phyla in mediating the Cd speciation transformation in the rice growth layer (Zeng et al., 2011). Anthropogenic management practices seriously altered the soil environments and further affected the Cd speciation variations. In plow layer soils, the decrease of soil pH and organic matter decomposition directly impeded the formation of the F-Oxi and F-Res fractions, and therefore the F-Aci and F-Red fractions accumulated gradually. However, soil pH and organic elements in plow pan soils indirectly influenced Cd speciation ratios through their adversely direct impact on the abundance of predominant bacterial taxa. The plow pan soil environment facilitated the formation of stabilized Cd fractions, which was consistent with the observations that soils with insufficient oxygen content and low ORP values could increase the immobilization of Cd (Khaokaew et al., 2011; Lin et al., 2020).

## Conclusion

This study investigated the Cd speciation distribution mechanisms regulated by soil properties and microbial communities between the plow layer and plow pan soils of Cd-contaminated paddy fields. The activated Cd speciation ratios were higher in plow layer soils and enhanced the Cd accumulative effect in rice grain, but stabilized Cd speciation ratios were elevated in plow pan soils. Soil physicochemical properties, dominant bacterial taxa, microbial diversities, and metabolic functions showed significantly different between the two soil samples. Differences in the soil environments of the plow layer and plow pan mediated diverse Cd speciation distribution. In plow layer soils, soil pH and organic elements decreased the formation of stabilized Cd speciation directly and resulted in the accumulation of activated Cd speciation, while the soil pH and organic elements indirectly hindered the transformation of activated Cd speciation in plow pan soils, and increased the stabilized Cd speciation *via* regulating the predominant bacteria. This study provides new insight into the effect of soil environments on Cd speciation transformation and will be helpful in bioremediating the Cd pollution of paddy field systems.

## Data Availability Statement

The datasets presented in this study can be found in online repositories. The names of the repository/repositories and accession number(s) can be found at: https://www.ncbi.nlm.nih.gov/, SRP291811.

## Author Contributions

XH, LB, and XL designed the study. PZ and HL conducted the experiment. YX and JG assisted with analysis tools of data. QL and HJ discussed the results and manuscript. XH and LH wrote the manuscript. All authors approved the final manuscript.

## Funding

This work was supported by the Shandong Provincial Natural Science Foundation (ZR2020QD120), the Project of Introducing and Cultivating Young Talent in the Universities of Shandong Province (Soil Erosion Process and Ecological Regulation; QC2019YY144), and the National Natural Science Foundation of China (41907032).

## Conflict of Interest

The authors declare that the research was conducted in the absence of any commercial or financial relationships that could be construed as a potential conflict of interest.

## Publisher’s Note

All claims expressed in this article are solely those of the authors and do not necessarily represent those of their affiliated organizations, or those of the publisher, the editors and the reviewers. Any product that may be evaluated in this article, or claim that may be made by its manufacturer, is not guaranteed or endorsed by the publisher.

## Footnotes

^1^http://zhoulab5.rccc.ou.edu/

^2^http://ieg2.ou.edu/MENA

^3^http://bioinfogp.cnb.csic.es/tools/venny/index.html
