# Supplementary material for: Cadmium Speciation Distribution Responses to Soil Properties and Soil Microbes of Plow Layer and Plow Pan Soils in Cadmium-Contaminated Paddy Fields
Source: Front Microbiol. 2021 Dec 3;12:774301. doi: 10.3389/fmicb.2021.774301 (PMC8679784; doi:10.3389/fmicb.2021.774301)
Supplement: Supplementary file 1 [file Table_1.DOCX]

## Supplementary Figures


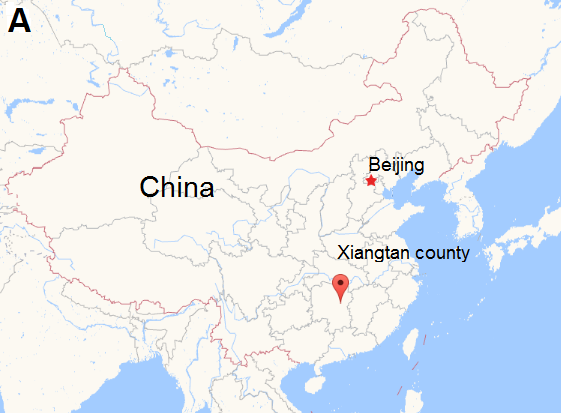

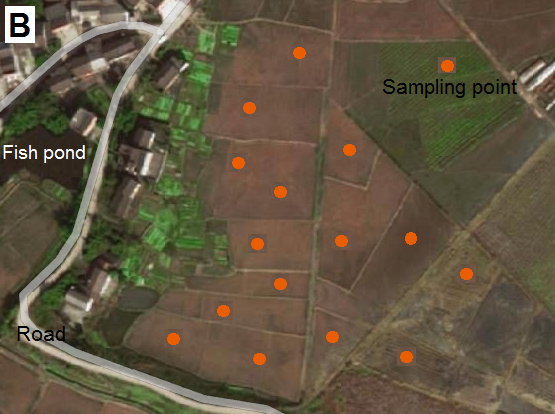


**Supplementary Figure 1.** (A) Location of Cd-contaminated paddy field investigated in this study and (B) basic information of sampling points.

**
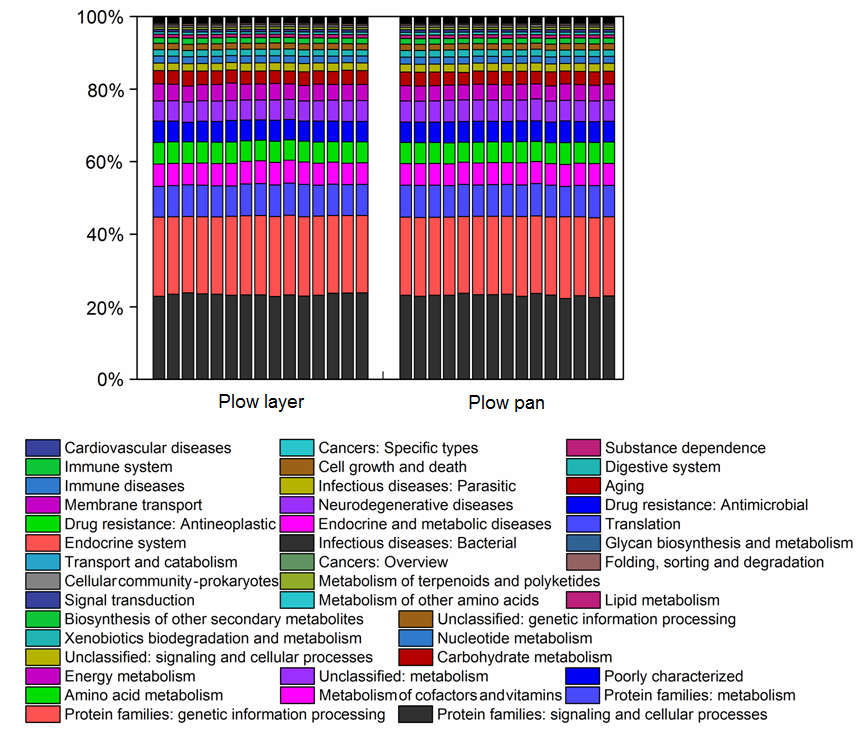
**

**Supplementary Figure 2.** Functional predictions of all of the samples using PICRUSt, comparing 40 level 2 KEGG orthology groups (KOs) represented in the data set between the plow layer and plow pan soil samples.

## Supplementary Tables

**Supplementary Table 1****.** Pearson correlation analysis between the Shannon index and Cd speciation ratio.

| Item | F-Aci ratio |  | F-Red ratio |  | F-Oxi ratio |  | F-Res ratio |  |
| --- | --- | --- | --- | --- | --- | --- | --- | --- |
|  | r | *P* | r | *P* | r | *P* | r | *P* |
| Plow layer | 0.353 | 0.196 | 0.014 | 0.959 | 0.075 | 0.790 | 0.249 | 0.370 |
| Plow Pan | -0.585 | **0.022** | 0.117 | 0.678 | -0.338 | 0.217 | 0.194 | 0.487 |

**Supplementary Table 2.** Topological properties of phylogenetic molecular ecology networks of microbial communities in the plow layer and plow pan soil samples.

| Network indexes | Plow layer | Plow pan |
| --- | --- | --- |
| Total nodes | 310 | 160 |
| Total links | 551 | 317 |
| R square of power-law | 0.893 | 0.665 |
| Average degree (avgK) | 3.962 | 3.555 |
| Average clustering coefficient (avgCC) | 0.269 | 0.337 |
| Average path distance (GD) | 8.305 | 4.089 |
| Geodesic efficiency (E) | 0.182 | 0.360 |
| Harmonic geodesic distance (HD) | 5.480 | 2.781 |
| Maximal degree | 20 | 15 |
| Nodes with max degree | OTU_192 | OTU_198 |
| Centralization of degree (CD) | 0.054 | 0.070 |
| Centralization of betweenness (CB) | 0.222 | 0.055 |
| Maximal stress centrality | 65590 | 3118 |
| Centralization of stress centrality (CS) | 1.306 | 0.231 |
| Nodes with max eigenvector centrality | OTU_33 | OTU_198 |
| Efficiency | 0.984 | 0.917 |
